# COVID-19 Associated Cardiovascular Disease—Risks, Prevention and Management: Heart at Risk Due to COVID-19

**DOI:** 10.3390/cimb46030124

**Published:** 2024-02-29

**Authors:** Andrew Kemerley, Abhishek Gupta, Mahesh Thirunavukkarasu, Monica Maloney, Sean Burgwardt, Nilanjana Maulik

**Affiliations:** Department of Surgery, Molecular Cardiology and Angiogenesis Laboratory, University of Connecticut School of Medicine, UConn Health, 263 Farmington Avenue, Farmington, CT 06030, USA; kemerley@uchc.edu (A.K.); abhgupta@uchc.edu (A.G.); mthirunavukkarasu@uchc.edu (M.T.); sburgwardt@uchc.edu (S.B.)

**Keywords:** SARS-CoV-2, ACE2, spike protein, myocardium, pneumonia, cytokines, long COVID

## Abstract

The SARS-CoV-2 (Severe Acute Respiratory Syndrome Coronavirus-2) virus and the resulting COVID-19 pandemic have had devastating and lasting impact on the global population. Although the main target of the disease is the respiratory tract, clinical outcomes, and research have also shown significant effects of infection on other organ systems. Of interest in this review is the effect of the virus on the cardiovascular system. Complications, including hyperinflammatory syndrome, myocarditis, and cardiac failure, have been documented in the context of COVID-19 infection. These complications ultimately contribute to worse patient outcomes, especially in patients with pre-existing conditions such as hypertension, diabetes, or cardiovascular disease (CVD). Importantly and interestingly, reports have demonstrated that COVID-19 also causes myocardial injury in adults without pre-existing conditions and contributes to systemic complications in pediatric populations, such as the development of multisystem inflammatory syndrome in children (MIS-C). Although there is still a debate over the exact mechanisms by which such complications arise, understanding the potential paths by which the virus can influence the cardiovascular system to create an inflammatory environment may clarify how SARS-CoV-2 interacts with human physiology. In addition to describing the mechanisms of disease propagation and patient presentation, this review discusses the diagnostic findings and treatment strategies and the evolution of management for patients presenting with cardiovascular complications, focusing on disease treatment and prevention.

## 1. Introduction

The global death toll from COVID-19 is estimated to be over 6.9 million as of September 2023 [1]. Poor patient outcomes in severe cases are commonly caused by the development of acute respiratory distress syndrome (ARDS), pneumonia, or septic shock [2,3]. However, systemic infection by SARS-CoV-2 virus impacts more than just the respiratory system and can cause both acute disease burden and long-term consequences in the cardiovascular system [4]. The global epidemiology of the virus has shown that patients with underlying cardiovascular diseases are disproportionately susceptible to increased morbidity and mortality from their illness [5]. Noteworthy pathological characteristics of the virus include the ability to upregulate inflammation by producing a cytokine storm and the capacity to directly infect myocardial cells [6]. The cardiovascular consequences of COVID-19 can be severe, and patients who are most at risk require enhanced monitoring and treatment to avoid adverse outcomes. Comprehending the effects of COVID-19 on the cardiovascular system in both adults and children enhances our knowledge of the infection processes and clinical outcomes.

The primary aims of this review were to describe how cardiovascular complications from COVID-19 manifest and review the proposed mechanisms by which these cardiovascular complications occur. The secondary aims of this review were to present the cardiovascular sequelae of infection and describe the medical management of these resultant illnesses. The tertiary aim of this review was to briefly discuss the roles, risks, and benefits of vaccination.

## 2. COVID-19 Classification and Structure

SARS-CoV-2, a member of the coronavirus family, causes the illness known as COVID-19. Coronaviruses are positive-sense single-stranded RNA viruses consisting of a nucleocapsid that possesses a spike protein [7]. This spike protein allows the virus to bind to specific receptors and infect cells within the body, leading to viral replication and proliferation of disease [8]. Sequencing analysis has shown that the virus is a member of the betacoronavirus (β-CoVs) family, to which the MERS and original SARS coronaviruses also belong. More specifically, SARS-CoV-2 belongs to the Sarbecovirus lineage of β-CoVs [9]. SARS-CoV-2 shares approximately 79.5% of its sequence identity with the virus SARS-CoV. However, the transmissibility of the initial SARS-CoV-2 variant, Alpha (B.1.1.7), is estimated to be ten times faster than that of the SARS-CoV virus, resulting in rapid spread that has contributed to the development of the pandemic [10,11].

Over time, the SARS-CoV-2 virus has continued to mutate, producing new disease variants characterized by differences in its spike glycoprotein [12]. Particular variants of concern include the beta, delta, and, most recently, omicron strains. The omicron variant has also spawned a variety of subvariants that are currently being monitored [13,14,15,16].

## 3. SARS-CoV-2 Interaction with Angiotensin Converting Enzyme 2 Receptor (ACE2)

Although the different strains of SARS-CoV-2 vary with respect to virulence and the severity of the symptoms induced, each strain has continued to propagate disease by the same mechanism. The SARS-CoV-2 coronavirus enters host cells by interaction of its spike glycoprotein with the host’s cell angiotensin-converting enzyme 2 (ACE2) receptor, sialic acid receptor, transmembrane serine protease 2 (TMPRSS2), and extracellular matrix metalloproteinase inducer (CD147). Catepsins B and L are also involved in the process of virus entry [17]. The ACE2 receptors convert Angiotensin II, a product of the renin-angiotensin-aldosterone system (RAAS), to Angiotensin 1-7. ACE2 receptors exist throughout the body, including in the pulmonary, gastrointestinal, renal, and cardiac tissues [18]. Consequently, the organs associated with each of these tissues may be damaged by infection. However, the presence of the ACE2 receptor is not predictive of which cells are most likely to be affected by the virus. Zhou L et al. reported that the level of ACE2 expression in the respiratory system is marginal and that the most significant levels of expression are observed in the kidney and intestinal tract; however, infection heavily damages the lungs to a greater extent [18]. Multiple studies have reported that SARS-CoV-2 requires co-factors to bind appropriately to ACE2 receptors, the most notable of which is transmembrane serine protease 2 (TMPRSS2). TMPRSS2 acts to proteolytically cleave the spike protein and activate it for binding [19,20,21,22,23,24,25,26,27]. Studies evaluating the extent to which the virus infects different organs based on the number of ACE2 cells available have demonstrated that the presence of additional co-factors makes the lungs and heart especially vulnerable to infection.

The mechanism by which SARS-CoV-2 enters the host cell via interactions with the ACE2 receptor and TMPRSS2 may also be a key explanatory factor for sex-related differences in the lethality of COVID-19 [9,13]. Although located on the X chromosome, the ACE2 receptor escapes X inactivation [28]. This observation suggests that the second X chromosome in the female sex can potentially confer protection against polymorphisms that render COVID-19 more aggressive in males [28]. ACE2 receptors are also influenced by estrogen, resulting in a shift of the ACE/ACE2 ratio towards the ACE2/ANG-1-7/MAS receptor axis, which may explain why women are more protected against severe COVID-19 outcomes [28]. TMPRSS2 is a testosterone-regulated gene, the greater expression of which in males may explain more severe COVID-19 outcomes [28].

**Figure 1:**
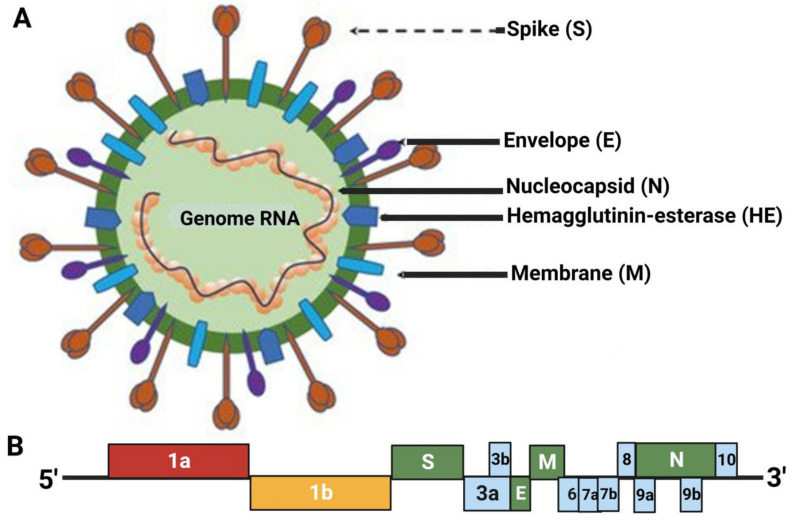
β-coronavirus particle and genome. (**A**) The β-coronavirus particle. β-coronavirus is an enveloped, nonsegmented, positive-sense single-stranded RNA virus genome in size ranging from 29.9 kb. The virion has a nucleocapsid composed of genomic RNA and phosphorylated nucleocapsid (N) protein, which is buried inside phospholipid bilayers and covered by the spike glycoprotein trimmer (S). The membrane (M) protein hemagglutinin-esterase (HE) and the envelope (E) protein are located among the S proteins in the virus envelope. (**B**) 5′ and 3′ terminal sequences of the SARS-CoV-2 genome. The gene order is 5′-replicase ORF1ab-S-envelope(E)-membrane(M)-N-3′. ORF3ab, ORF6, ORF7ab, ORF8, ORF9ab, and ORF10 are located at the predicted positions shown in the picture. 1a, 1b, 3a, 3b, 6, 7a, 7b, 8, 9a, 9b, 10 in the picture represent different ORF genes. *(*Figure 1 was adapted from Weiss et al. and reprinted with permission) [30].

The presence of a highly acidic environment may also limit the ability of the virus to infect the gastrointestinal and renal organ systems [29,30,31]. The β-coronavirus particles and genome structure are shown in Figure 1 [30]. Coronaviruses have the largest known RNA genome (30–32 kb) and encode two large genes (ORF1a and ORF1b). The structural proteins are spike (S), envelope (E), membrane (M), and nucleocapsid (N) proteins. The M-protein is the central player in virus assembly, the S-protein is present on the surface of the virus, and the E-protein is important for the virus–host cell interaction [32].

**Figure 2:**
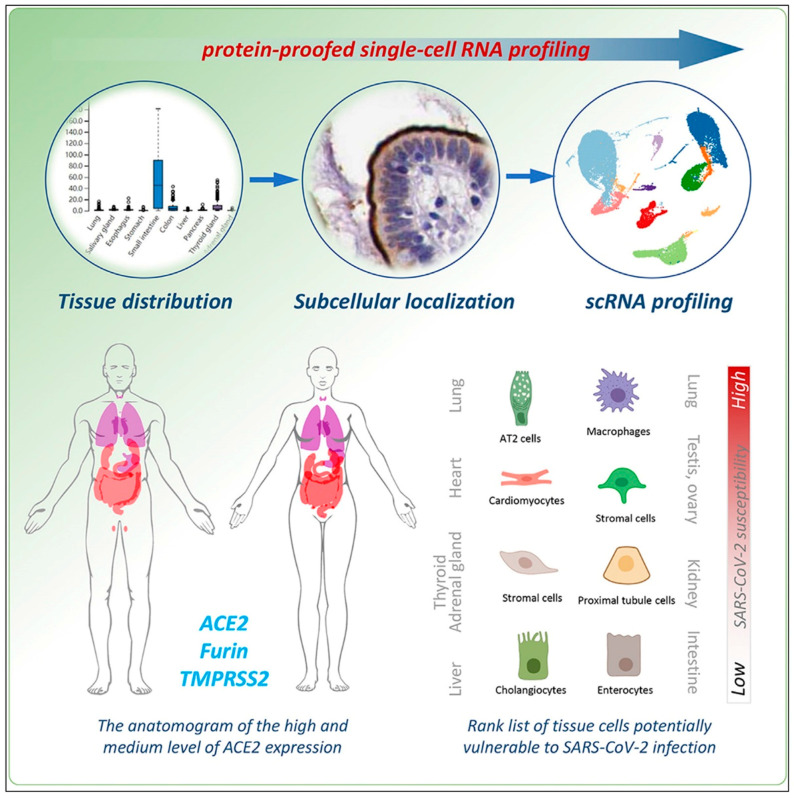
Through protein-proofed single-cell RNA (pscRNA) profiling of ACE2 receptors and co-factors TMPRSS2 and Furin, 10 potential SARS-CoV-2 targets were identified and ranked in order of susceptibility. The origin of the primary cell targets identified were the lung, heart, and gonads. There is a discrepancy between ACE2 expression and COVID-19 symptoms: the map represents the organs susceptible based on clinical evidence. The protein expression level of ACE2, protein location within the cell, co-expression of infection co-factors like Furin and TMPRSS2, possible entry routes and environments for virus entry and propagation, and the mRNA level of ACE2 present within the cell itself all play a role. The red color in this figure visually demonstrates that the body tissues most susceptible to infection (e.g., lung) are a result of a combination of these factors, which allow virus propagation, not the actual expression of ACE2 protein within the tissues. (Figure 2 was adapted from Zhou et al. and reprinted with permission) [18].

The factors cathepsins B and L also participate in viral entry [17]. The expression of these factors in endothelial cells may explain the endothelial dysfunction seen in COVID-19 patients. Figure 2 summarizes the work done by Zhou, L. et al. to elucidate cells susceptible to the deleterious effects of SARS-CoV-2 by RNA profiling the relative expression of specific molecular targets in various tissue types [18]. Some of the commonly proposed therapies to treat COVID-19 patients include tocilizumab, colchicine, chloroquine/hydroxychloroquine, azithromycin, and famotidine. All of these potential therapeutic medications have been shown to improve endothelial function, which may provide additional evidence that endothelial dysfunction caused by SARS-CoV-2 contributes to its virility [33].

## 4. Clinical Presentation of COVID-19

COVID-19 presents with a wide variety of symptoms, including headache, nausea/vomiting, fever, sore throat, nasal congestion, rhinorrhea, dizziness, muscle cramps, and fatigue [34]. Severe cases of COVID-19 can result in ARDS, pneumonia, and septic shock [34]. In less severe COVID-19 cases, the goals of care are symptomatic management and patient isolation. In more severe infections or infections in vulnerable populations, including the elderly, obese, and those with hypertension (HTN) or diabetes mellitus (DM), it is essential to minimize the exacerbation of underlying conditions [35]. HTN and DM are the most prevalent comorbidities in hospitalized COVID-19 patients and should be appropriately monitored [36].

In the context of cardiovascular disease, COVID-19 may present with elevated troponin levels, ST-elevation myocardial infarction (STEMI), arrhythmias, and fulminant myocarditis [37]. Disease severity is also increased in patients with underlying CVD, including HTN, heart failure, arrhythmia, and coronary artery disease (CAD) [38]. In some studies, it has been shown that the case fatality rate of hospitalized patients infected with COVID-19 was 10.5% in those with underlying CVD and 6% in those with underlying HTN [39,40]. In comparison, for the same period, the overall case fatality rate for all patients (not stratified by comorbidity) was much lower at 2.3% [40]. Studies have also shown that hypertensive patients have a higher mortality risk from COVID-19 than who are normotensive patients [36]. A study conducted in Wuhan, China, in early 2020 found that myocardial injury, indicated by elevated troponin levels, was positively associated with fatal outcomes in patients hospitalized due to COVID-19 (37.50% vs. 7.62% for patients without myocardial injury) [41,42,43,44,45,46]. The authors also showed that patients with underlying CVD who were infected with COVID-19 and suffered myocardial injury had a mortality rate of 69.44% [5,45]. The prevalence of myocardial injury due to COVID-19 infection is estimated to be approximately 20%, and such injuries could be a critical mediating factor in patient prognosis [5]. Furthermore, there is a positive correlation between increased cardiac troponin levels and disease severity, suggesting that troponin is a clinically relevant biomarker for a more severe disease course [47]. Finally, myocardial injury is associated with a higher incidence of complications such as ARDS (58.5% vs. 14.7%), acute kidney injury (AKI), and coagulopathy [36].

The severity of COVID-19 is characterized by three phases: mild, moderate, and severe pneumonia [36]. According to Azevedo et al., 80% of patients resolve after phase 1; approximately 15% of patients enter the second phase at approximately the 10th day of symptom onset, and approximately 5% of those infected move into the third phase ofsevere pneumonia [36]. The third phase is characterized by a hyperinflammatory response, where the inflammatory mediators IL-6, IL-2, and TNF-alpha are pathologically upregulated, potentially resulting in a cytokine storm [36]. Although approximately 80% of COVID-19 cases resolve spontaneously, 5% evolve into the third phase of the disease [2,36]. This review describes the potential mechanisms by which the inflammatory response occurs and propagates. However, it must be noted that the upregulation of inflammatory mediators, even before the development of a cytokine storm, contributes to the onset of fulminant myocarditis and damaged cardiac tissue in these patients [23,35]. The development of hyperinflammatory syndromes after infection is of particular concern in the pediatric population [14]. Severe infection in this group is relatively rare (accounting for 1% of hospital admissions), with most infected patients presenting with no or mild symptoms [14]. However, some patients with severe disease develop a hyperinflammatory response termed the Multisystem Inflammatory System in Children (MIS-C) [14,48,49,50,51,52,53,54,55,56]. Although novel in its etiology, this syndrome presents similarly to Kawasaki’s disease, with symptoms that include fever, fatigue, diffuse erythematous rash, non-purulent conjunctivitis, diarrhea, nausea, and vomiting [53,57]. The treatment and management of this condition are similar to those of patients with Kawasaki disease, with intravenous immunoglobulin (IVIG) and corticosteroids used to mitigate the hyperinflammatory environment.

## 5. Findings from Cardiovascular Imaging after COVID-19 Infection

Various cardiac manifestations may result from the SARS-CoV-2 infection. Evaluation of patients via echocardiography has demonstrated myocardial injury with left ventricular (LV) dysfunction, specifically diastolic dysfunction with decreased left ventricular ejection fraction and right ventricle (RV) dilatation with or without dysfunction [58,59,60]. There is also evidence that RV afterload increases due to lung damage and that elevated troponin levels are associated with worse RV dysfunction [58,59]. In addition, pulmonary acceleration time is shorter, with worsening RV fractional change, lower systolic tricuspid lateral annular velocity, and tricuspid annular plane excursion, suggesting an increased RV afterload [58]. The diversity of findings suggests that multiple processes may explain the effect of COVID-19 on cardiac tissues. A meta-analysis summarizing the most common cardiac abnormalities observed via both echocardiography and cardiac magnetic resonance (CMR) showed that many patients with COVID-19 have elevated pulmonary arterial systolic pressure (PASP) [61]. This analysis also revealed that lung damage caused by the virus could lead to increased pulmonary artery pressure and, ultimately, RV dysfunction [61]. Although LV dysfunction and LV global longitudinal strain (LV-GLS) are commonly reported, RV dysfunction appears to be more prevalent and is likely the source of observed elevated troponin levels [61,62]. A systematic review of echocardiographic findings demonstrated that systolic LV dysfunction was observed in <10% of the patients with abnormal findings [58]. Evaluation of RV strain patterns could be beneficial, in addition to measuring cardiac biomarkers to evaluate disease prognosis. RV strain patterns are impaired early, even in mild forms of the disease [59]. Furthermore, cardiac injury, LV-GLS, and RV-GLS are independent predictors of mortality [63]. Electrocardiograms are also clinically relevant for patient assessment because T-wave inversion detection can assist in identifying stress cardiomyopathy and myocarditis [63].

**Figure 3:**
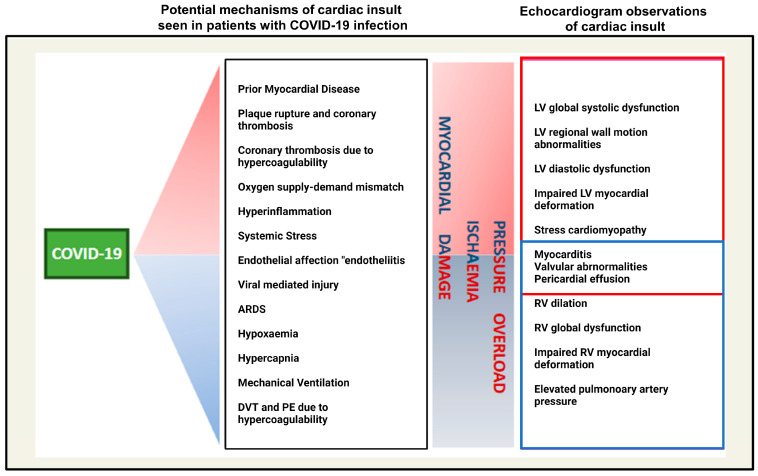
Manifestations of COVID-19 on echocardiogram and potential mechanisms by which they occur. The following abbreviations are used in the image: RV—Right ventricular, LV—Left ventricular, ARDS—acute respiratory distress syndrome, DVT—deep vein thrombosis, PE—pulmonary embolism. (Figure 3 was adapted from Carrizales et al. and reprinted with permission) [58].

Overall, echocardiography and CMR precisely assessed myocardial injury and cardiac complications in patients with COVID-19. However, patients may not always have access to such imaging modalities, nor are they commonly used or indicated in clinical practice. In such cases, cardiac biomarkers, including troponin and creatine kinase, and ultrasound imaging may be readily available diagnostic tools that can be used to identify cardiovascular complications [61] (Figure 3).

## 6. Pathophysiology of COVID-19 in Impacting the Cardiovascular System

**Figure 4:**
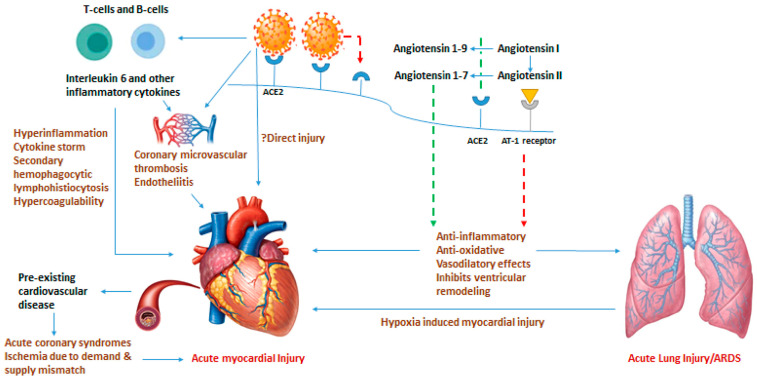
Potential pathophysiological pathways of COVID-19 in impacting the cardiovascular system. These include direct injury, inflammation, hypoxia, and pre-existing CVD. The following abbreviations are used in the image: ACE—angiotensin-converting enzyme (ACE), ARDS—acute respiratory distress syndrome. Green dashed line—Positive effect; Red dashed line—negative effect. (Figure 4 was adapted from Bavishi et al. and reprinted with permission) [47].

**Figure 5:**
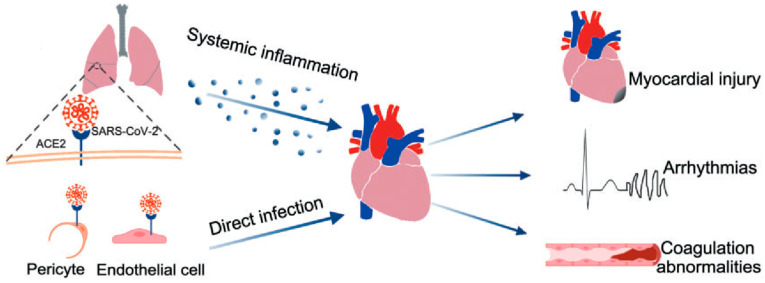
Model of acute cardiac injury in COVID-19. Direct infection of pericytes and endothelial cells, as well as systemic inflammation, promote myocardial injury, arrhythmias, and coagulation dysfunction such as disseminated intravascular coagulation (DIC). (Figure 5 was adapted from Liu et al. and reprinted with permission) [2].

COVID-19 may damage the heart via several mechanisms, including cardiac myocyte binding and endothelial cell damage. Cardiac myocytes possess a large number of ACE2 receptors, second only to lung tissue cells [18]. This makes these cells highly susceptible to SARS-CoV-2 binding, entry, and infection [18]. The virus damages cells, leading to dysregulation and dysfunction that can result in myocardial injury and downstream inflammatory effects [47]. Additionally, endothelial cell damage has also been implicated in the pathophysiology of COVID-19 (Figure 4). Endothelial cells in both the heart and vasculature are targets of cardiovascular and systemic complications [64]. Some studies have suggested that ACE2 receptors are located within endothelial cells and potentiate infection in a direct manner, similar to myocytes [65,66,67,68,69,70,71]. Further research has also postulated that pericytes, the cells that surround endothelial cells, may be the actual mediators of disease because they also possess ACE2 receptors [72]. The direct mechanism (s) of infection involving endothelial cells and pericytes require further investigation; however, evidence for indirect mechanisms of COVID-19-induced endothelial cell damage and dysfunction, causing cardiac tissue damage, is better supported (Figure 5). Endothelial cells may be damaged by systemic processes, including reactive immune and inflammatory cytokine-mediated responses induced by COVID-19. This cytokine storm is discussed in the following section [17,73,74].

## 7. Cytokine Storm

**Figure 6:**
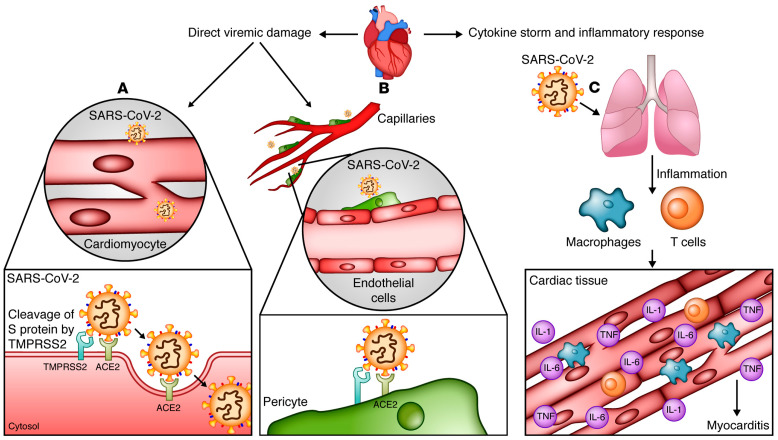
(**A**): SARS-CoV-2 spike (S) protein binds to TMPRSS2 on cardiomyocytes. TMPRSS2 cleaves the S protein and facilitates its activation and binding to cell-entry receptor angiotensin converting enzyme 2 (ACE2) on the surface of cardiomyocytes. (**B**) In addition, pericytes, those cells adjacent to and in commune with cardiomyocytes to maintain functionality and maintenance of the endothelial cell layer of the myocardium also express surface ACE2 receptors. Injury of these cells by SARS-CoV-2 results in endothelial cell dysfunction. (**C**) SARS-CoV-2 also triggers a cytokine storm and systemic inflammatory responses which contributes to cardiac tissue damage and myocarditis. Adapted by Farshidfar et. al. and reprinted with permission [23].

**Figure 7:**
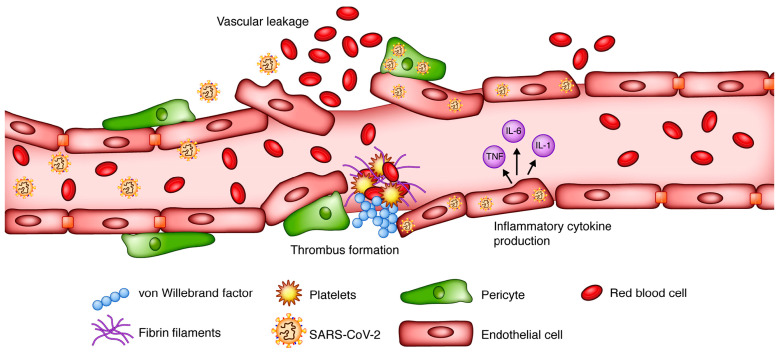
SARS-CoV-2 infection disrupts the cellular integrity of endothelial cells, resulting in vascular leakage that promotes hypercoagulable conditions through von Willebrand factor-mediated thrombus formation. It additionally activates cytokine secretion by endothelial cells, which contributes to further exacerbating these inflammatory effects. (Figure 7 was adapted from Farshidfar et al. and reprinted with permission) [23].

As discussed above, COVID-19 has pathologic effects on multiple organ systems. Its presence in the body initiates a systemic inflammatory response triggered by the immune system. An imbalance in the response of Type 1 and Type 2 helper T cells and the prolonged nature of the immune response can lead to a cytokine storm [75]. Prolonged inflammation can lead to myocarditis and direct cardiac tissue damage [75]. Furthermore, downregulation of ACE2 receptors due to binding of the virus decreases the protective effects of converting angiotensin 2 to angiotensin 1-7, resulting in pro-inflammatory, pro-oxidative, and vasoconstrictive effects, which induce hyperinflammatory responses and increase the risk of coagulopathy [3]. Additional inflammation due to circulating cytokines can potentiate atherosclerotic plaque instability and lead to Type I myocardial infarction [3]. This mechanism of atherosclerotic plaque instability combined with previously described endothelial cell damage increases the likelihood of acute myocardial infarction (Figure 6 and Figure 7).

## 8. Hypoxia and Underlying Comorbidities

COVID-19 is a disease process that can directly damage pneumocytes. Pneumocyte infection taxes the respiratory and lung parenchyma’s capacity to oxygenate blood, which leads to hypoxia. This hypoxic insult can lead to oxidative heart stress and ultimately result in cardiac injury and cell death, leading to type II myocardial infarction due to demand ischemia [3]. Additionally, this hypoxic state can result in the buildup of intracellular calcium, further damaging the myocardial tissue [3]. The immune response initiated by COVID-19 creates an inflammatory state that can potentially exacerbate the underlying CVD, HTN, and DM through the pathways described above. Microvascular thrombosis and endothelitis can also cause ischemia and pathological inflammation. Such ischemia may also be exacerbated by increased physiological demands and lung injury incurred during acute infection [36,39,75].

## 9. Treatment, Management, and Prevention of COVID-19

Given the multifaceted symptomology of COVID-19, ranging from patients with asymptomatic infection to those with severe disease burden requiring mechanical ventilation, the overall treatment strategy for COVID-19 may vary across patient populations. For patients with a mild illness, treatment primarily consists of the isolation and care of symptoms [76]. For those with a more severe disease course, the infection may cause severe viral pneumonia. In such cases, the initial treatment typically focuses on maintaining adequate tissue oxygenation [77,78]. This task is accomplished by supplemental oxygenation and mechanical lung-protective ventilation to prevent the development of ARDS [77]. The use of systemic corticosteroids in patients with confirmed COVID-19 pneumonia who require oxygen therapy due to hypoxia decreases mortality [76,77,78,79,80,81]. Antibiotics are not routinely started because bacterial coinfection with COVID-19 is rare; however, a broad-spectrum antibiotic regimen is used if a superimposed infection is suspected [82,83,84].

As previously stated, a hyperinflammatory environment may occur in patients with a severe disease course, increasing the risk of small and large vessel thrombosis [85,86,87]. To mitigate hypercoagulability in those patients at risk, prophylactic anticoagulation is administered according to individual patient factors (most commonly subcutaneous heparin) [88,89,90].

Substantial evidence supports antiviral therapy in the form of paxlovid (ritonavir-boosted nirmatrelvir) and veklury (remdesivir) for patients with a high risk of progression to severe illness caused by COVID-19 [91,92,93]. These high-risk populations include patients over the age of 50 years, unvaccinated patients, and patients with specific medical conditions such as CVD, DM, or obesity [94,95]. During the pandemic, metformin, ivermectin, and fluvoxamine were repurposed as potential therapies to mitigate and/or prevent severe COVID-19-related illness. In a double-blind, randomized control trial, none of these drugs significantly reduced hypoxemia, emergency department visits, hospitalization, or death associated with COVID-19. Overall, treatment strategies remain individualized for specific patients, with a focus on preventing progression to the severity of illness requiring hospitalization.

One potential option to prevent progression to severe disease is the management of other comorbidities like DM, hypertension, hyperlipidemia, smoking, or obesity. Cardiac rehabilitation programs are useful adjuncts for preventative medicine to reduce the risk of COVID-19-induced cardiac damage. Cardiovascular exercise has been shown to have cardioprotective benefits that affect myocardial oxygen demand, endothelial function, self-reliance tone, coagulation and coagulation factors, inflammatory indicators, and the creation of collateral coronary arteries [96,97,98].

The unprecedented rapid development of COVID-19 vaccinations was a remarkable accomplishment for the biomedical community and was estimated to have saved 20 million lives during the first year of global deployment [99,100]. The World Health Organization (WHO) approved ten COVID-19 vaccines for global use, comprising eight distinct vaccine formulations. These include inactivated, messenger RNA (mRNA), adenovirus vector-based, and adjuvanted protein vaccines. Due to their high cost, chilling requirements, distribution, and logistics, mRNA vaccines are used primarily in the United States and Europe, with relatively limited use in developing nations [99,100,101]. On the other hand, adenovirus vector-based vaccines are more stable than mRNA vaccines and require no refrigeration, resulting in their more widespread utilization in developing nations. Global immunization rates vary, with over 70% of eligible individuals in the United States, the majority of other developed nations being fully immunized, and less than 15% of individuals in the African region being fully immunized [99,100,101,102,103,104].

Population immunity continues to be monitored closely, and the comparative durability of various vaccines is a topic of continued interest. The mRNA vaccines induce strong short-term immunity via an antibody response; however, these titers wane several months after the vaccine is administered and decline further around 8 months [101,105,106]. Adenoviral-based vaccines induce a lower initial antibody titer; however, the immune response remains high and consistent for approximately 8 months, in contrast to mRNA vaccines [71,101,107,108,109]. Waning immunity from mRNA vaccines is correlated with increased breakthrough infections in vaccinated people, as reported in Provincetown, Massachusetts, in the summer of 2021 [110]. Overall, the COVID-19 pandemic appears to have transitioned to an endemic phase. Current vaccines appear to be less effective at neutralizing the omicron variant; however, protection against severe disease remains [101].

## 10. Vaccine-Related Myocarditis

A small proportion of people who received the mRNA-based vaccine developed mild myocarditis. In these cases, the vaccine is hypothesized to be an immune trigger for subsequent subsequentmyocardial inflammation [26]. Rare but similar vaccine-triggered inflammatory events have been documented, such as in the case of the smallpox vaccine in the 1940s, with naive individuals undergoing their first vaccination having the highest risk of occurrence [111,112,113]. One descriptive study found that 1626 cases of myocarditis were reported out of 192,405,448 doses of COVID-19 vaccine. The study concluded that the risk of myocarditis after receiving an mRNA-based COVID-19 vaccine increased across multiple age and sex strata and was highest after the second vaccination dose in adolescent males and young men [26]. Notably, the clinical course proved to be mild in a small portion of patients who developed myocarditis, with preserved cardiac function, rapid recovery (typically within 6 days), and no short-term complications [114]. Both thrombosis and myocarditis are far more likely to occur following COVID-19 infection than after vaccination [101].

Globally, the introduction of various COVID-19 vaccines has resulted in a significant decline in COVID-19-related morbidity and mortality, and all approved vaccines have demonstrated benefits that transcend potential risks (including myocarditis) in all age groups [114,115,116,117].

## 11. Chronic Implications of COVID-19 on the Cardiovascular System

Because the pandemic started less than four years ago, it is too early to fully understand the chronic, long-term implications of infection in people, including any effects on the respiratory and cardiovascular organ systems. However, it is clear that chronic conditions resulting from COVID infection will continue to have a measurable impact on global health. In an effort to improve clarity and consensus among healthcare providers and the global healthcare community, the WHO described long-COVID as a diagnosis of exclusion, with the development or continuation of symptoms 3 months after the initial occurrence of COVID-19 symptoms and persistence of symptoms for at least 2 months. Symptoms of long COVID may include fatigue, shortness of breath, and cognitive impairment/dysregulation [118].

Approximately 144.7 million people globally suffered from the symptoms of long COVID in 2020 and 2021 [119]. However, a dearth of research has limited the extent of the current knowledge on the emerging and chronic effects of COVID-19. For example, in a systematic literature review that assessed over 1000 articles addressing the acute and chronic implications of COVID-19 on the cardiovascular system, only one study followed the same cohort of patients for >12 weeks to assess the response to therapeutics after COVID-19 infection [120]. Clearly, more research is needed to assess how long COVID manifests and to evaluate potential therapeutic strategies.

Of the data that we currently have regarding this topic, the existence of a long COVID was estimated to be experienced by 6.2% of individuals with prior symptomatic infection by a meta-regression performed by Hanson et al. [119] The impact of long COVID on the cardiovascular system is particularly likely to play a role in the global healthcare landscape, as survivors continue to live with disease sequelae. In studies specifically examining cardiovascular function after COVID infection, such as the cohort study of German patients (*n* = 100) by Putmann et al., 60% of patients who had recently recovered from COVID had ongoing myocardial inflammation after recovery, and 78% of recently recovered patients were found to have lower left ventricular ejection fraction and higher left ventricle volumes when compared to controls [121]. Additionally, epidemiological studies have shown that SARS-CoV-2 causes a 15-fold increase in the incidence of myocarditis and pericarditis [122]. This evidence of clinically significant changes in cardiovascular health after COVID infection is incredibly relevant to our evaluation of patients in the coming years as we continue to observe the ways in which long COVID affects the population. It is paramount that complications in the cardiovascular health of patients infected with SARS-CoV-2, particularly those who had severe disease or suffered from persistent symptoms, should be assessed with the history of their infection in mind so that the long-term consequences of this disease can be further defined.

## 12. Summary and Conclusions

Given the unprecedented initial manifestation and subsequent pandemic caused by the novel SARS-CoV-2 virus, continued evaluation of viral transmission and the mechanisms by which its deleterious health effects manifest are critical. COVID-19 is often considered to be strictly a respiratory virus; however, its systemic effects are likely to lead to long-term health complications both involving and independent of the respiratory system. Understanding the systemic pathophysiology of this virus, including its effects on the cardiovascular system, is essential to improve our knowledge of this and future viral pathogens.

As highlighted in this review, COVID-19 can severely impair the cardiovascular system, with dramatic dysfunction in myocardial tissue. Various mechanisms contribute to this impairment, including binding of the virus to cardiac myocytes and endothelial cells, induction of a cytokine storm, and resultant tissue hypoxia. The adverse effects of the virus on the cardiovascular system may be exacerbated in the context of pre-existing comorbidities, including CVD, HTN, and DM. The diverse presentation of cardiac abnormalities in COVID-19 patients supports the multifaceted virulence of this microbe in the cardiovascular system. For this reason, potential cardiovascular complications from this disease should be assessed in infected patients to expedite their discovery and allow for timely treatment. It is also essential to evaluate the long-term effects of the virus on overall health because we do not yet fully understand its natural course.

Treatment and management of severe infections with antiviral therapies and anti-thrombolytic agents are necessary. Prevention of the disease through vaccination is a crucial tool to combat the virulence of COVID-19, which remains a global presence despite decreasing infection rates. Although myocarditis is a rare side effect known to be associated with mRNA vaccines, the potential risks of vaccination, including myocarditis, remain markedly lower than those of COVID-19 infection. Therefore, the scientific community must continue to educate the public that vaccines are safe and effective.

As the world adapts to the reality of COVID-19 as an endemic disease and regains a semblance of life before the onset of the pandemic, it is valuable to reflect on one of the most challenging periods in human history. COVID-19 has challenged every aspect of our lives, including our scientific understanding of the world. We are forever indebted to the healthcare workers and scientists who have created novel therapies and vaccines to combat this disease. Without the development of such vaccines and therapies for COVID-19, we would most certainly have experienced more illnesses and deaths. However, this public health crisis, of a magnitude that has not been seen for over 100 years, also highlighted persistent disparities and inequities in global healthcare systems. Efforts to understand this disease and its effects on population health should continue, with this inquiry being used as a fulcrum to improve healthcare delivery. Investigation and enhanced preparation will allow us to address future pathogenic threats with greater scientific knowledge, thereby improving health outcomes and mitigating the infrastructural damage inflicted by global pandemics.
